# Impact of foliar fertilization on apple and pear trees in reconciling productivity and alleviation of environmental concerns under arid conditions

**DOI:** 10.1080/19420889.2019.1565252

**Published:** 2019-01-07

**Authors:** Meisam Zargar, Antonina Tumanyan, Elizaveta Ivanenko, Anna Dronik, Natalya Tyutyuma, Elena Pakina

**Affiliations:** a Department of AgroBiotechnology, Institute of Agriculture, RUDN University, Moscow, Russia; b Department of Fruit and Berry Crops, Near-Caspian Scientific Research Institute of Arid Agriculture, Astrakhan, Russia

**Keywords:** Foliar nutrition, apple, pear, drought, high stress

## Abstract

Drought and heat stress are significant factors limiting fruit crop yield in arid conditions. Foliar fertilization is a common practice of supplying fruit crop production with mineral nutrients, especially under limited soil nutrient availability conditions. To evaluate potential effectiveness of the foliar application of macro-, micronutrient and growth regulators on dynamics of physiological parameters of the pear and apple cultivars under abiotic stresses, three–year experiments were carried out under arid conditions at the Russian Research Institute of Arid Agriculture during the 2015–2017 growing seasons. It has been revealed that foliar nutrition reduces the negative influence of heat stress, stabilizes the functional state of plants, thereby enhancing resistance to drought. During the most severe drought periods of vegetation, under the influence of foliar nutrition, there was a significant increase in the total water content (TWC), relative leaf turgidity (RLT) and water retention capacity (WRC); and also index of leaf water deficiency (LWD) was improved as compared to the non-treated control. All foliar treatments involving the macro-, micronutrient and growth regulators significantly enhanced fruit crop yield of pear and apple varieties over the control, yield enhancement was obtained 2.7–22.0 t ha^−1^ for the Talgar beauty (pear variety), 2.2–19.3 t ha^−1^ for the Renet Symirenko (apple variety), and 1.6–10.5 t ha^−1^ for the Starkrimson (apple variety). The most effective treatments for water consumption coefficient (WCC) were plantafol and speedphol. The results suggest that foliar plantafol and speedfol could be used as part of an efficient, sustainable fertilizer program for apple and pear trees for maintaining or improving fruit quality, productivity, and avoiding negative efficacy of abiotic stresses.

## 1. Introduction

Perennial fruit trees are constantly exposed to various abiotic stresses during their lifetime and this has an adverse effect on fruit crop yield. The need for effective nutrient management is crucial for optimizing fruit crop production [1], and enhance plant ability to tolerate various environmental stresses. Nonetheless, most fruit growers apply large amounts of chemical fertilizers to the soil more than what the tree actually requires, which could result in the excess nutrients being carried away by surface runoff causing eutrophication in water bodies [2,3]. Foliar sprays have also been employed as a significant tool to meet tree nutrient demand. This fertilization method is more target-oriented and environmentally friendly since the nutrients are applied in controlled quantities [4].

Drought and heat stresses are major abiotic factors capable of reducing photosynthetic efficiency by curtailing leaf expansion, thus, causing premature leaf senescence, in several regions of the world, inclusive of western Asia and southern Russia [5]. Plants often experience drought stress due to the erratic distribution of precipitation [6,7]. Hence, low moisture and drought stress are demonstrated as the predominant environmental factors, reducing plant productivity in many arid and semi–arid areas, which are intensively affected by climate changes [8]. Heat stress has the potential to cause serious disturbances in plant growth and development which may be due to membrane disruptions, metabolic changes and generation of oxidative stress [9].

Tree fruit yield and quality can depreciate as a response to deficiencies in any mineral nutrient and foliar application of some elements such as nitrogen (N), calcium (Ca), potassium (K), and boron (B), is closely tied to fruit quality. In fruit tree production, apple and pear growers can apply fertilizers whenever soil analysis results exhibit insufficient nutrient amounts to feed the plant for the obtainment of optimum yields. Macronutrient and micronutrient foliar fertilizers are typically complementary to soil applications and are applied to quickly diminish nutrient deficiencies that appear suddenly, such as those caused by excessive vegetative growth or nutrient imbalances caused by improper applications of fertilizers or soil amendments. Micronutrients are vital and applied in small quantities, nevertheless are essential for various biochemical and physiological processes of plant growth and development [10]. This study focuses on developing foliar spray programs and strategies that increase nutrient phytoavailability [10], consequently reducing nutrient requirements and minimizing the release of potentially deleterious elements into the environment, and that allow safe tank mixing of multiple nutrients, thus reducing the number of sprays required per season [11,12].

Environmental concerns such as heat and drought stresses typically do not affect plants independently, but in several different combinations under field conditions and the influence of joint stress factor action does not equate the sum of separate stress factor effects [13]. During the plant phonological stages vulnerable to drought effects, moisture stress occurs in various forms such as; pre-flowering water deficit (regions of South America); grain–filling (post–anthesis) water deficit (Mediterranean regions) and continuous water deficit [14]. Foliar fertilization has progressed over time, following many decades of research and development, an indispensable tool for the sustainable agricultural approach and is of supreme commercial importance internationally [15,16]. In addition to achieving more practical experience regarding the optimum stages of foliar fertilizer application, little is known about the possibilities of improving foliar nutrient penetration into leaf or fruit tissues [16]. In many orchards, macronutrients and especially micronutrients are leaf-applied routinely to prevent the deficiencies of these essential elements [17]. Moreover, it has become evident that foliar nutrients can regulate flowering, fruit yield and fruit quality. An extensive experiment was undertaken to study the potential effect of foliar fertilization on apple and pear trees to ameliorate productivity and alleviate environmental stresses. The main objective of the study was to investigate the principal aspects of the foliar application of macro–, micronutrients and growth regulators with regards to crucial factors of fruit cropping systems, apple and pear trees under conditions of low moisture availability and high summer temperatures.

## 2. Materials and methods

### 2.1. Site description and soil

The recent study was carried out during the cropping seasons of 2015–2017 in the arid conditions of southern Russia, Astrakhan region. The experimental orchard was located at the Scientific Research Institute of Arid Agriculture, Astrakhan Region, Russia. The research station is located at 42°58′ N, 47°28′ E and 130 m altitude above sea level.

Soil samples were randomly collected from each replication in the form of 15–cm deep soil cores (20 cores per replication), before planting and fertilizer application. The soil samples were dried at approximately 50°C, ground, and analyzed using standard methods by Clemson University Agricultural Service Laboratory (Clemson, SC, USA).

### 2.2. Experimental design and treatments

Varieties of Apples (Starkrimson and Renet Simirenko) and Pears (Talgar Beauty) were planted in a commercial setting at 2.4 m × 4.8 m and 2.7 m × 5.5 m spacing receptively on a loamy soil with 1.5% organic matter and pH of 7.1 in spring 2009. Trees were 2.5 to 3.5 m tall at the time of the experiment, the orchard was irrigated by drip irrigation. A drip irrigation system 50 m long, was installed. The hydraulic performance of emitters was based on water flow, uniformity coefficient, application efficiency, and water losses through deep percolation.

Complete randomized block design with three replications was employed for arranging the six investigated foliar fertilization treatments consisting of Nitroammophoska, Boroplus, Plantafol, Speedfol, Megafol and non-treated a control, whereas each block was represented by a single tree. The study described in this paper utilized individual trees from the larger study. Selection of these trees was based on uniform tree size as determined by similar trunk diameters [18,19].

Foliar fertilizations were applied using a carbon dioxide (CO_2_) propelled sprayer with TJ60-8002VS flat fan nozzles (TeeJet Technologies, Wheaton, IL, USA) at 240 L ha^−1^ and no surfactant was used.

Concentrations for aqueous solutions of the following agrochemicals used in this study were in the range recommended for foliar applications in orchard. The aqueous solutions of following agrochemicals used in this study were outlined in Table 1. Other cultural practices were similar to those of commercial apple and pear orchards in the fruit tree growing region of Astrakhan.

**Table 1.** Active ingredient of agrochemicals used in experiments.

Agrochemicals	Type	Active Ingredient W/W %
Nitroammophoska	Macronutrient	NH_4_H_2_PO_4_ 20.0%
		NH_4_NO_3_ 20.0% KCL 20.0%
Boroplus	Micronutrient	Boron 11.0%
Plantafol	Macronutrient	Total nitrogen 20.0% (Nitrate −4, Ammonia −2, Urea-14)
		Phosphoric acid 20.0%
		Soluble potassium 20.0%
		Trace elements: B-0.02%, Fe * 0.01%, Mn * −0.05%, Cu * −0.005 (* – chelates in the form of EDTA)
Speedfol	Growth Stimulants (with	Amino Acid 33.5%
	anti-stress activity)	MgO 2.7%
		CaO 6.7%
Megafol	Growth Stimulants (with	Amino Acid 28.0%
	anti-stress activity)	Total Nitrogen 3.0%
		Amide 2.0%
		Incl. organic 1.0%
		Soluble potassium (K_2_O) 8.0%
		Organic carbon (C) 9.0%

Agrochemicals were used based on recommendations of the manufacturing companies. Foliar applied solutions were performed in following phases:

- Nitroammophoska twice: at growing fruit and beginning of ripening
- Boroplus twice: at stages of beginning of flowering and Growing fruit
- Plantafol four times: at stages of pink bud, beginning of flowering, growing fruits and beginning of ripening
- Speedfol four times: at green tip, full bloom, pink bud and beginning of flowering
- Megafol twice: at stages of before budding and growing fruits

### 2.3. Data recording

In drought conditions, the indicators characterizing the plant’s functional state are the general moisture content of the leaves, water–holding capacity, turgidity and moisture deficiency.

For the study of the efficiency of non-nutritive nutrition, the physiological state of apple and pear trees were analyzed at the second day of June, July and August. For this purpose, in the morning hours 3–fold repeat samples of 10 leaves from the middle tier of the crown were taken, packed in bags, delivered to the laboratory and weighed. Total water content was determined on crude and dry mass. To determine the water deficit and the relative turgidity, samples were reweighed after being oven–dried at 70°C for 24 hours’ saturation for a percentage of the crude mass of the leaf.

Water retention capacity of leaves was determined by the loss of water over four hours of wilting. Entire data were computed by calculating the average of three replicates for each feature [20].

All measurements were executed on electronic laboratory of the Precisa (Swiss brand). Total water content (TWC), leaf water deficiency (LWD), relative leaf turgidity (RLT) and water retention (WRC) were calculated using the formulas:

$$TWC=100\left(M-M_{2}\right)/M$$

$$LWD=100\left(M1-M\right)/\left(M_{1}-M_{2}\right)$$

$$LWD=100\left(M-M{\;}_{2}^{\;}\right)/\left(M_{1}-M_{2}\right)$$

$$WRC=100\left(M-M_{3}\right)/\left(M-M_{2}\right)$$

Where M is the mass of the fresh sample; M_1_ – mass of the sample after 24 h of saturation; M_2_ – sample weight after drying; M_3_ – mass of the sample after 4 h of wilting. Entire data were computed by calculating the average of three replicates for each feature [20]. The coefficient of water consumption (WCC) was calculated with regards to the formula:

$$WCC=\frac{W,m^{3}/ha}{Y,t/ha}$$

Where W is the total water consumption, including precipitation during the growing season, the difference in the supply of productive moisture between the beginning and the end of vegetation, and the irrigation rate for the season; Y – productivity, t ha^−1^.

### 2.4. Statistical analysis

Experiments were laid out in a randomized complete block design. All statistical analyzes were performed using SAS and MSTAT-C computer programs. The data were analyzed by One–Way Analysis of Variance (ANOVA). Mean separations were performed by Least Significant Difference (LSD) test. Differences at P ≤ 0.05 were illustrated as significant.

## 3. Results and discussion

### 3.1. Climate conditions of survey area

Climate conditions during 2015–2017 were extremely unfavorable in terms of precipitation coupled with the high mean daily air and soil temperature, moisture deficiency resulted during experimental seasons. In June, the temperatures soared to a range of 36.1–40.3°C, in July it was 38.7–42.3°C, and in August 39.1–40.4°C. Precipitation fell by 3.6–11.7 mm (June), 3.9–23.0 mm (July), and 9.1–24.1 mm (August).

**Figure 1.**
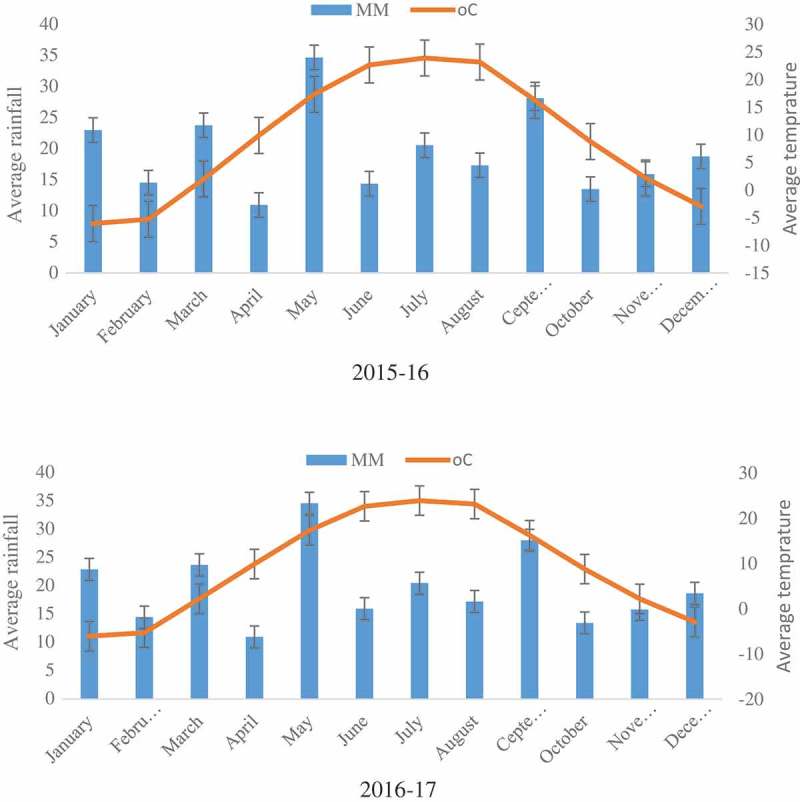
Metrological data during the course of present studies (Source: Near–caspian scientific research institute of arid agriculture).

During experimental years, meteorological data regarding temperature and rainfall was obtained from Near-Caspian Scientific Research Institute of Arid Agriculture (Figure 1).

### 3.2. Dynamics of total water content

The weather conditions that prevailed during the study were extremely unfavorable in terms of precipitation and also with the high mean daily air and soil temperatures, moisture deficiency ensued considerably during experimental seasons. All treatments including the application of micro-, macronutrients and growth regulators significantly (P < 0.05) increased total water content (TWC) in comparison with the control, showing a low coefficient of variation (3.12 to 9.70%) (Table 2). Furthermore, the highest indexes of total water content of leaves were recorded in June with the value of 60.3–64.9% for apple varieties and 59.7–62.6% for pear variety. During the growing season, this index favorably diminished in August. However, TWC was the least as 52.5–58.7% (apple varieties) and 53.9–56.3% (pear). In all cases, by using foliar fertilization, leaf water content of pear and apple varieties was higher than that of the non-treated control (Table 2).

**Table 2.** Dynamics of the total water content of leaves under the influence of various foliar fertilization (%) – mean values 2015–2017.

	Pear			Apple					
	Talgar Beauty			Starkrimson			Renet Simirenko		
Treatments	June	July	August	June	July	August	June	July	August
Control	58.0d	56.7d	52.9d	57.5c	56.7d	53.8e	58.8d	56.7d	52.0e
Nitroammophoska	59.7cd	58.7b	55.4b	64.9a	57.3c	54.4d	64.2a	60.0a	55.0c
Boroplus	62.6a	58.1bc	55.5b	62.4b	59.4a	56.6b	63.1b	59.5b	58.7a
Plantafol	60.4c	57.8c	56.3a	62.5b	58.5b	55.2c	60.8c	57.5c	54.3d
Growth stimulants Speedfol	61.0b	60.9a	53.9c	62.2b	59.2a	57.4a	60.3c	59.9b	57.2b
Growth Stimulants Megafol	61.7b	58.7b	55.4b	62.3b	58.9b	55.5c	60.4c	57.5c	52.5c
LSD _0.05_	2.51	0.98	2.37	4.61	0.60	0.62	3.02	0.95	1.58
CV %	3.12	6.21	5.84	3.55	4.49	6.99	9.70	8.19	3.85

Leaf water content is one of the most important characteristics of the plant’s water balance. Well-known physiological action of growth regulators is their ability to induce fruit tree tolerance against environmental stresses [1]. Relative water content plays an important role in regulating stomatal conductance and hence the photosynthetic rate of the plant. High temperature and drought stress induce morphological [21] as well as physiological and biochemical changes in plants. Combined action of these stresses induces changes in water relations [22], leaf water content, decrease in photosynthesis [23], hormonal changes and cell membrane thermostability [24].

### 3.3. Dynamics of leaf water deficit

As shown in Table 3, all foliar treatments altered the leaf water deficit of pear and apple varieties, the lowest water deficit during the summer was stably observed in all varieties with speedfol (growth regulator) (P < 0.05), with the value of 12.0–19.0% for apple cultivars and 13.5–18.9% for pear. LWD in non-treated control attained 18.7–28.2% for apple varieties and 18.5–28.0 for pear variety (P < 0.05) (Table 3). In general, by applying foliar nutrition, LWD was significantly reduced below values attained in the non-treated control (Table 3). In addition to speedfol, the dynamics of LWD were positively influenced by other foliar nutrition including Nitroammophoska, Boroplus, Plantafol and Megafol. Hence, this determines the functional stability of plants in the drought growing season, under the influence of experimental treatments (Table 3). Mentioned findings were typically similar to those reported previously [25,26]. LWD triggers production of the phytohormone abscisic acid (ABA), which in turn causes stomatal closure and the induction of abiotic stress-responsive genes [27].

**Table 3.** Dynamics of water deficiency of leaves under the influence of foliar treatments (%) – mean values 2015**–**2017.

	Pear			Apple					
	Talgar Beauty			Starkrimson			Renet Simirenko		
Treatments	June	July	August	June	July	August	June	July	August
Control	18.5a	23.2a	28.0a	19.7a	20.6a	27.3a	18.7a	19.9a	28.2a
Nitroammophoska	17.4b	20.0cd	25.4c	16.5c	20.0a	26.3b	16.8c	17.1c	27.2b
Boroplus	14.5c	19.0d	23.3d	19.2a	19.8ab	22.8d	14.7d	13.9d	15.0e
Plantafol	13.3d	20.7c	27.5b	17.9b	18.1b	26.1b	13.9e	19.2a	20.9c
Growth stimulants Speedfol	13.5d	22.5b	18.9e	12.0d	16.4c	17.9e	13.0e	16.5cd	19.0d
Growth Stimulants Megafol	17.2b	20.9c	24.7cd	12.2d	18.4b	24.1c	17.5b	18.7b	27.0b
LSD _0.05_	0.68	1.56	3.32	1.13	1.57	1.73	1.56	1.01	2.07
CV%	2.99	8.59	2.29	3.80	8.21	9.52	6.19	2.88	3.31

In fact, drought is a meteorological phenomenon associated with the lack of rainfall in a certain period of time. These periods are long enough to cause soil moisture depletion and water deficit stress along with the reduction of water potential in plant tissues. The inadequacy of the quantity and distribution of available water during the plant growth period has reduced the apparent full genetic potential of the plant, and its destructive effect on the yield and final income of the farmer is well known [28,29].

Means in columns followed by the same letter are not significantly different at P *= *0.05; CV = Coefficient of variation.

Means in columns followed by the same letter are not significantly different at P *= *0.05; CV = Coefficient of variation.

### 3.4. Dynamics of relative leaf turgidity

The water status of leaves is a key property associated with turgidity, photosynthesis, and respiration. Stressed plants tend to lose turgor recovery capacity, especially after sunset, with the addition of water stress [30,31]. The relative turgidity technique has been widely used to analyze leaf water status, and on occasion to estimate the total water potential of leaves [32]. It was found out that the relative turgidity in July and August was lower than in June, as when thermal stress increases, relative leaf turgidity (RLT) enhances (Table 4). Through the use of foliar fertilizers, turgidity values of both apple and pear varieties were significantly (P < 0.05) ameliorated in drought and high temperature conditions since they were affected by almost all experimental treatments (Table 4).

**Table 4.** Dynamics of relative leaf turgidity under the influence of foliar treatments (%) – mean values 2015**–**2017.

	Pear			Apple					
	Talgar Beauty			Starkrimson			Renet Simirenko		
Treatments	June	July	August	June	July	August	June	July	August
Control	86.6c	83.4c	81.0d	85.7c	77.7e	76.6c	78.9d	78.9d	74.0d
Nitroammophoska	91.2a	85.6b	89.5b	86.7b	79.4d	76.4c	82.6c	80.2c	75.7c
Boroplus	91.5a	85.9b	91.0a	86.2b	84.0a	80.9a	86.7a	82.5a	82.4a
Plantafol	91.2a	85.2bc	89.5b	86.1b	80.1c	78.4b	83.5bc	79.9c	78.8bc
Growth stimulants Speedfol	88.3b	90.5a	86.5c	88.8ab	81.2b	80.0a	84.5b	82.4a	79.7b
Growth Stimulants Megafol	90.9a	86.7b	89.2b	89.4a	81.7b	79.6ab	83.1c	81.0b	79.4b
LSD _0.05_	1.81	2.26	4.71	3.02	3.44	1.03	4.14	3.10	2.00
CV%	1.99	9.10	1.89	6.33	7.09	4.55	8.02	9.11	3.37

The highest level of RLT from June to August was observed for Starkrimson apple variety (79.6–89.4%) when megafol (growth regulator) was applied. Aqueous solution of boroplus had the best RLT increase (P < 0.05) for Rennet Simirenko (apple variety) and Talgar Beauty (pear variety) with values of 82.4–86.7% and 85.9–91.5% respectively. These results are in agreement with an earlier study [33] in which foliar–applied growth regulator significantly improved leaf turgidity potential. Overall, using foliar application of nutrition desirably enhanced RLT of pear and apple varieties over the non–treated control (Table 4). Leaf wilting is a symptom of turgor loss, hence, it is an important simple phenotypic expression of a critical stage in plant water status under drought stress.

Foliar application of fertilizers can improve leaf turgidity status while increasing the plant’s resistance to environmental stresses, hence, foliar nutrient is one of the most commonly used methods to deliver nutrients by spraying water-soluble fertilizers to plant foliage. Through spraying, the plant absorbs nutrients into ionic forms, via the foliage. Suitable nutrition of plants is one of the important aspects of plant health and productivity. Leaf spray application is used as a source of nutrients for the plant, which provides better nutrition, enhance fruit quality and increase resistance to the various abiotic stresses as thermal and drought. The relative turgidity of a leaf is a measurement of its actual water content relative to its maximal water holding capacity at full turgidity. RLT provides a measurement of the water deficit of the leaf, and also indicates a certain degree of environmental concerns expressed under drought and heat stress [34].

Means in columns followed by the same letter are not significantly different at P *= *0.05; CV = Coefficient of variation.

### 3.5. Water retention capacity

Physiological stability of plants under the influence of drought conditions was illustrated by the water retention capacity of leaves. The water retention capacity (WRC) of leaves exhibited in the Table 5 determines the ability of plants to withstand prolonged droughts. This is explained by the increase in the amount of bound water in the cells of the leaves and the osmotic pressure of the cell sap [35]. In June, WRC index, depending on the foliar treatment used, was 12.5**–**20.5% for the Talgar Beauty variety, 11.5**–**13.7% for Starkrimson and 10.9**–**23.3% for the Renet Simirenko variety (Table 5).

**Table 5.** Dynamics of water retention capacity of leaves (%) – mean values 2015**–**2017.

	Pear			Apple					
	Talgar Beauty			Starkrimson			Renet Simirenko		
Treatment	June	July	August	June	July	August	June	July	August
Control	11.3e	10.1d	15.3c	9.8d	8.6c	8.7e	9.9d	10.5d	11.5c
Nitroammophoska	12.5d	14.5b	18.9b	12.9b	10.0b	9.6d	14.1b	12.3c	15.1b
Boroplus	17.6b	13.0c	19.8b	13.7a	12.5a	11.1c	14.1b	9.5e	15.9b
Plantafol	14.3c	10.6d	18.8b	11.6c	10.6b	17.0a	23.3a	19.6a	18.2a
Growth stimulants Speedfol	12.9d	9.8d	18.8b	11.7c	12.8a	14.0b	14.4b	15.0b	15.3b
Growth Stimulants Megafol	20.5a	21.5a	26.4a	11.5c	10.8b	14.0b	10.9c	9.4e	12.0c
LSD _0.05_	2.68	2.07	3.69	2.39	1.32	2.16	3.45	2.01	1.12
CV%	3.02	2.29	7.89	5.19	6.09	5.55	2.18	1.09	4.90

In July, WRC index for Talgar beauty (pear), Starkrimson (apple) and Rennet Simirenko (apple) under wilting diminished to 9.8**–**21.5%, 10.0**–**12.8% and 9.4**–**19.6% respectively, so that is probably an adaptive mechanism to changes in air temperature and humidity in the summer. In August, during the period of fruit filling, WRC again enhanced to values of 18.8**–**26.4% for the pear variety, and 9.6**–**18.2% for apple varieties. The best results for this index in apple trees was obtained with the use of plantafol (17.0**–**23.3%) (P < 0.05) and 20.5**–**26.4% (P < 0.05) was achieved for pear variety by using megafol (Table 5). A number of reports are available indicating the role of nutrients in alleviating various abiotic stresses [24]. When plants are affected by stresses, they increase their osmolyte concentration so that water absorption continues under stress conditions. Among organic osmolytes, proline is probably the most abundant and most common compatible soluble material which accumulates [25,36]. Heat stress can cause scorching of leaves and twigs, sunburns on leaves, branches and stems, leaf senescence and abscission in plants [37].

Means in columns followed by the same letter are not significantly different at P *= *0.05; CV = Coefficient of variation.

### 3.6. Fruit yield

The fruit yield is the main determinative factor that proves the accuracy of the selection of a specific fertilization program. Analysis of foliar nutrition effectiveness on response of fruit crop yield in specific weather conditions during the summer indicated that productivity of the various apple and pear cultivars was favorably enhanced when foliar nutrition treatments were performed. All foliar treatments involving the macro–, micronutrient and growth regulators significantly (P < 0.05) enhanced fruit crop yield of pear and apple varieties over the non-treated control, showing a low coefficient of variation (2.82 to 7.92%) (Table 6).

**Table 6.** Effect of treatments on the yield of pear and apple varieties – mean values 2015–2017.

Treatments	Productivity. kg/tree	Yield. t ha^−1^	Compared to Control	
			t ha^−1^	%
Talgar Beauty (Pear)				
Control	46.1f	14.4f		
Nitroammophoska	54.6e	17.1e	2.7	18.8
Boroplus	72.5c	22.7c	8.3	61.0
Plantafol	116.6a	36.4a	22.0	152.8
Growth stimulants Speedfol	94.9b	29.6b	15.2	105.6
Growth Stimulants Megafol	62.d	19.7d	5.3	36.8
LSD _0.05_	19.68	5.89		
CV%	7.92	5.17		
Renet Simirenko (Apple)				
Control	34.3f	28.6f		
Nitroammophoska	47.2c	39.3c	10.7	37.4
Boroplus	41.8d	34.8d	6.2	21.7
Plantafol	57.5a	47.9a	19.3	67.5
Growth stimulants Speedfol	51.0b	42.5b	13.9	49.6
Growth Stimulants Megafol	37.0e	30.8e	2.2	7.7
LSD _0.05_	16.57	11.09		
CV%	2.82	4.14		
Starkrimson (Apple)				
Control	21.0d	17.4d		
Nitroammophoska	20.9d	17.8d	0.4	2.2
Boroplus	27.4b	22.8b	5.0	28.1
Plantafol	33.6a	28.0a	10.2	57.3
Growth stimulants Speedfol	33.9a	28.3a	10.5	59.0
Growth Stimulants Megafol	23.3c	19.4c	1.6	9.0
LSD _0.05_	11.98	1.89		
CV%	5.05	4.80		

The yield enhancement with the use of foliar application was 2.7–22.0 t ha^−1^ for the Talgar Beauty variety, 2.2–19.3 t ha^−1^ for the Renet Symirenko variety and 1.6–10.5 t ha^−1^ for the Starkrimson variety. As shown in Table 6, plantafol and speedfol were the most effective aqueous solutions for all varieties of apple and pear.

The improvements in plant growth and enhancement of fruit yields could be attributed to large increases in plant nutrition after foliar applications. Foliar fertilization might be more beneficial for ameliorating plant growth when soil and climate condition are not suitable, and plants are under stresses. Overall, it can be concluded that an appropriate foliar nutrient supply is crucial to attain high yields in fruit trees.

Environmental stresses trigger a series of changes in the plant, which can occur in the growth, physiological and chemical composition structure of the plant. These changes may inhibit growth thereby reducing plant yield. Abiotic stress leads towards morphological, physiological, biochemical and molecular changes which negatively affect plant growth and productivity [38,39]. The effectiveness of spring applied foliar urea sprays for fruit trees is controversial. Some researchers have reported that foliar nutrition applied is equally or more effective than soil nutrition applications in improving fruit set and subsequent fruit size and yield [10]. Results obtained under our study were consistent with the findings of Kumar et al. [40] who stated that foliar application of macro–and micronutrients resulted in the highest fruit set and yield of apple. Foliar applications of microelements before full blossom also increased fruit set and yield of pear [41,42].

### 3.7. Water consumption coefficient

The fruit crop yield is an effective determining factor on the value of the water consumption coefficient (WCC). Application of growth regulators and water soluble mineral fertilizers containing available forms of nutrients significantly contributes to the stimulation of growth processes. We accounted for the water use coefficient for apple and pear varieties influenced by the various foliar fertilization treatments. The highest water consumption for the formation of a unit of fruit produce was attained in the non-treated control, with values of 206.6–320.7 m^3^/t for apple cultivars and 340.9 m^3^/t for pear (Table 7). Foliar application of macro–, microelements and growth regulators in this study determined an increase in yield, and correspondingly, a reduction in WCC by 123.4–191.9 m^3^/t, 201.7–294.3 m^3^/t and 134.9–287.1 m^3^/t (P < 0.05) for apple varieties Renet Simirenko, Starkrimson and pear variety Talgar Beauty respectively (Table 7).

**Table 7.** Coefficient of water consumption of pear and apple varieties according to the variants of the experiment (m^3^/t) – mean values 2015**–**2017.

	Pear	Apple	
Treatments	Talgar Beauty	Starkrimson	Renet Simirenko
Control	340.9a	320.7a	206.6a
Nitroammophoska	287.1b	292.1b	150.4d
Boroplus	216.3d	250.4c	169.8c
Plantafol	134.9f	203.9d	123.4f
Growth stimulants Speedfol	165.8e	201.7d	139.0e
Growth Stimulants Megafol	249.2c	294.3b	191.9b
LSD _0.05_	8.16	7.08	10.99
CV%	13.61	2.83	6.10

The most effective treatments on water use coefficient were plantafol and speedphol, such that water consumption values for the formation of one-ton fruit produce were 123.4–139.0 m^3^, 201.7–203.9 m^3^ and 134.9–165.8 m^3^ for apple varieties Renet Simirenko, Starkrimson and the pear variety Talgar Beauty respectively, which once again proves the positive and significant efficacy of foliar fertilizing on the water status of apple and pear trees. Apple trees require a balanced and adequate supply of macro – and micronutrients for growth and yield. There are many evidence associating improvement of water use efficiency [43] and water consumption coefficient with nutrient supply. However, in this study, foliar application of nutrients triggered to improvement in water use coefficient for apple and pear varieties through enhancing plant resistance to the abiotic stresses. Optimum economic and sustainable apple yields can only be achieved with judicious application of fertilizers [44,45].

Means in columns followed by the same letter are not significantly different at P *= *0.05; CV = Coefficient of variation.

Means in columns followed by the same letter are not significantly different at P *= *0.05; CV = Coefficient of variation.

## 4. Conclusion

Abiotic stress such as drought and extreme temperatures are key environmental factors that may affect morphology, phenology and plant biochemistry at all levels of organization. Our study demonstrates an effective approach that can significantly reduce negative influence of heat stress and stabilizes the functional state of plants, thereby increasing resistance to drought conditions. Foliar fertilization strategies have a pivotal role in determining the quality of horticultural products, and can achieve higher nutrient use efficiencies while reducing environmental impacts and potentially enhancing consumer health benefits.
